# Prediction models for compassion fatigue in nurses: A protocol for systematic review and critical appraisal

**DOI:** 10.1371/journal.pone.0342524

**Published:** 2026-09-15

**Authors:** Zifeng Li, Xiaojing Zhou, Luhuan Yang, Zuyang Xi, Huiqin Liu, Yuanzhi Fu, Xiaojuan Zhang

**Affiliations:** 1 Department of Traditional Chinese Medicine, The First College of Clinical Medical Science, Three Gorges University/Yichang Central People’s Hospital, Yichang City, Hubei Province, China; 2 Department of Pediatrics, The First College of Clinical Medical Science, Three Gorges University/Yichang Central People’s Hospital, Yichang City, Hubei Province, China; 3 Faculty of Medicine and Health Sciences, Universiti Putra Malaysia, Serdang, Selangor, Malaysia; 4 Department of Nursing, the First College of Clinical Medical Science, Three Gorges University/Yichang Central People’s Hospital, Yichang City, Hubei Province, China; 5 Hubei Key Laboratory of Ischemic Cardiovascular Disease, Yichang City, Hubei Province, China; 6 Department of Nursing, Gansu Health Vocational College, Lanzhou City, Gansu Province, China; Alexandria University Faculty of Nursing, EGYPT

## Abstract

**Introduction:**

Nurses are routinely exposed to high emotional demands associated with illness, disability, and death. Prolonged exposure may deplete nurses’ capacity for compassion, contributing to compassion fatigue. Compassion fatigue not only erodes emotional resources but may also adversely affect professional functioning and mental health. To date, several studies have developed prediction models for nurses’ compassion fatigue, but the methodological quality of these studies remains uncertain.

**Aims:**

This systematic review will comprehensively summarize published prediction models for nurses’ compassion fatigue and describe their key characteristics, predictors, performance, and risk of bias.

**Methods:**

Using a prespecified search strategy, we will systematically search seven databases: PubMed, Web of Science, Cochrane Library, Embase, CINAHL, PsycINFO, and CNKI. Two reviewers will independently conduct study selection, data extraction, and quality assessment according to prespecified inclusion and exclusion criteria. The data extraction form will be developed in accordance with the CHARMS checklist, and the risk of bias of the included models will be assessed using the PROBAST tool. Results will be presented in tables to facilitate qualitative comparisons across models. The systematic review will be reported in accordance with the PRISMA 2020 statement. The protocol has been registered in INPLASY (registration number: INPLASY202530051).

**Conclusion:**

This review will identify and compare existing models for predicting compassion fatigue in nurses and will inform future model development, validation, and application.

## Introduction

Compassion is central to nursing. It lets nurses understand patients’ emotions and build trust with them [1]. When nurses show more compassion, doctor–patient conflicts may decrease. Patients may feel better and cooperate more. Compassion also raises nursing quality and benefits nurses’ health [2,3]. However, heavy workloads, such as frequent shifts and emergency care, drain nurses’ compassion. Stressful environments, like tense doctor–patient relationships, add to this burden. Repeatedly seeing patients’ suffering or death can cause compassion fatigue (CF) [4]. CF is a state nurses reach after long exposure to patients’ pain. It leads to less compassion and emotional exhaustion [5]. CF includes occupational burnout and secondary traumatic stress (STS) [6]. Burnout means less enthusiasm for work, exhaustion, and sometimes self-doubt [7]. STS happens when nurses face trauma at work. It can cause symptoms like anxiety and insomnia [8].

Compassion fatigue is now common among nurses worldwide. Its reported prevalence exceeds 50% [9]. A systematic review [10] of 156 studies (63,254 nurses) reported pooled mean scores for burnout (26.81) and secondary traumatic stress (25.88), indicating moderate levels. Data from 18 studies (5,007 nurses) showed moderate compassion fatigue (mean score: 53.78). The consequences of compassion fatigue are profound for nurses, healthcare organizations, and society. Nurses may suffer from depression, anxiety, and sleep disorders. They may also have somatic symptoms, such as headache and gastrointestinal dysfunction [11,12]. Professionally, compassion fatigue can reduce job satisfaction and increase clinical errors. It can also raise intention to leave [13,14]. Early identification and timely interventions are critical. They help protect nurses’ health and maintain healthcare quality.

Many factors influence compassion fatigue among nurses. These include demographic, psychological, and social or environmental characteristics. Demographic factors include gender, years of nursing experience, marital status, and educational background [15,16]. Psychological factors include psychological sensitivity, resilience, and professional identity [17–19]. Social and environmental factors include department, working hours, and working environment [20–22]. Identifying individual risk factors differs from developing multivariable prediction models. Such models combine several predictors to estimate an individual’s risk of compassion fatigue. They offer greater clinical utility through risk stratification and early screening. This enables targeted interventions for high-risk populations. Some studies have developed prediction models, but findings vary substantially [23–25]. For example, Zou Mei et al. used a logistic regression model with five predictors. It achieved sensitivities and specificities of 79.28% and 71.43%, respectively [23]. Liang developed a linear regression model with four predictors and a cut-off of 147.5. Sensitivity and specificity were 82.1% and 72.9%, respectively [24]. Wang Junjie et al. developed an eight-factor logistic regression model but did not report sensitivity or specificity [25]. These differences in statistical methods, predictors, and performance metrics show the heterogeneity among models for predicting compassion fatigue.

In summary, compassion fatigue is a pressing occupational health concern in nursing. Its high prevalence and many consequences make it a research priority. Many studies have identified risk factors for compassion fatigue. Yet the development and validation of multivariable prediction models remain inconsistent. These models are essential for screening, risk stratification, and early intervention. This study will systematically review published prediction models for compassion fatigue among nurses. It will evaluate their methodological quality and clinical applicability. Through rigorous appraisal and comparison, the review will identify models suitable for clinical implementation. For example, useful models could be part of hospital workforce early-warning systems. The review will also highlight models needing further validation and refinement. Its findings will provide an evidence base for future model development, validation, and implementation. They will guide research priorities and resource allocation in this field.

## Methods and analysis

This protocol follows the Preferred Reporting Items for Systematic Reviews and Meta-Analyses Protocols (PRISMA-P) guidelines (S1 Appendix) [26]. Data will be extracted in accordance with the Critical Appraisal and Data Extraction for Systematic Reviews of Prediction Modelling Studies (CHARMS) checklist (S2 Appendix) [27]. The Prediction Model Risk of Bias Assessment Tool (PROBAST) will be used to assess the risk of bias and applicability of the included prediction models (S3 Appendix) [28]. Finally, the review will be reported in accordance with the Preferred Reporting Items for Systematic Reviews and Meta-Analyses (PRISMA) 2020 statement (S4 Appendix) [29]. The protocol has been registered in the International Platform of Registered Systematic Review and Meta-analysis Protocols (INPLASY) (https://inplasy.com/inplasy-2025-3-0051/).

### Eligibility criteria

Table 1 presents the inclusion and exclusion criteria, developed in accordance with the PICOTS framework, across six domains: population, intervention/index test, comparator, outcomes, timing, and setting [30].

**Table 1:** Eligibility criteria for the systematic review framed according to the PICOTS system.

Item	Inclusion criteria
Population	licensed/registered nurses
Index	Models developed to predict or to validate compassion fatigue in nurses
Comparator	Not applicable
Outcome	Compassion fatigue
Timing	No limits
Setting	Hospital

### Population

#### Studies involving licensed/registered nurses will be eligible.

Intervention: All prediction models developed to predict compassion fatigue in nurses will be eligible, regardless of whether internal or external validation was performed.

### Outcome

Commonly used instruments to assess compassion fatigue include the Compassion Fatigue Self Test Scale (CFSTS), the Professional Quality of Life Scale (ProQOL), and the Compassion Fatigue Short Scale (CFSS) [31–33]. The CFSTS comprises two dimensions (compassion fatigue and burnout) with a total of 40 items. Figley subsequently revised the scale by adding a compassion satisfaction dimension, increasing the total number of items to 66. Cronbach’s alpha values for compassion satisfaction, burnout, and compassion fatigue were 0.87, 0.90, and 0.87, respectively [31]. The ProQOL contains 30 items across three dimensions: compassion satisfaction, burnout, and STS. Cronbach’s alpha values for the three dimensions were 0.88, 0.75, and 0.81, respectively [32]. The CFSS includes two dimensions (secondary trauma and burnout) with 13 items, and its Cronbach’s alpha is 0.90 [33]. Given that other instruments are also used to measure compassion fatigue, no restrictions will be placed on the measurement tools in this review. All studies reporting prediction models for compassion fatigue will be included, and the outcome definitions, measurement instruments, and reference criteria used to ascertain compassion fatigue will be summarized in the final table. The primary outcome is compassion fatigue, with prediction models for burnout and STS also included as related outcomes.

### Timing

There is no limit on the predicted time point, and any study predicted at any time will be included.

### Setting

All nursing practice settings, including but not limited to hospitals, community care, home care, hospice care, and long-term care facilities.

### Exclusion criteria

Studies will be excluded if they: a) involve nursing students, physicians, or other healthcare personnel (i.e., populations other than licensed/registered nurses); b) assess compassion fatigue using measurement instruments without established reliability and validity evidence; c) are not peer-reviewed (e.g., conference abstracts); d) have no accessible full text; e) do not report key information required to extract or evaluate prediction models (e.g., predictors and model performance measures); f) are published in languages other than English or Chinese.

### Types of studies and limits

No restrictions will be placed on study design. The search will cover the period from database inception to October 31, 2025.

### Search methods for the identification of studies

#### Information sources.

We will conduct a comprehensive search across seven databases (PubMed, Web of Science, the Cochrane Library, Embase, CINAHL, PsycINFO, and CNKI) and screen the reference lists of included studies to identify additional eligible studies.

### Search strategy

The search strategy will have three stages. First, a pilot search in PubMed will examine the characteristics of relevant records, such as titles, abstracts, and keyword patterns. Based on these results, we will consult nursing experts to finalize search terms and build the Boolean search string. Second, two researchers will independently run the searches across the databases using the specified strategies. Third, we will screen reference lists of included studies through backward citation searching. The PubMed search strategy appears in Table 2. Preliminary search strategies for all databases are in S5 Appendix.

**Table 2:** Full search string for the database “Pubmed”.

PubMed Search Strategy		
1. Population	Subject Headings	“Nurses”[MeSH Terms] OR “Nursing”[MeSH Terms]
	Keywords	“Nursing Personnel”[Title/Abstract] OR “Personnel, Nursing”[Title/Abstract] OR “Nurse”[Title/Abstract] OR “Nursings”[Title/Abstract]
2. index prediction model	AND	
	Subject Headings	“Predictive Learning Models”[MeSH Terms] OR “Risk Factors”[MeSH Terms] OR “Clinical Decision Rules”[MeSH Terms] OR “Nomograms”[MeSH Terms] OR “ROC curve”[MeSH Terms] OR “Predictive value of tests”[MeSH Terms] OR “Observer variation”[MeSH Terms]
	Keywords	“Learning Model Predictive”[Title/Abstract] OR “Model Predictive Learning”[Title/Abstract] OR “Predictive Learning Model”[Title/Abstract] OR “Predictive Models Machine”[Title/Abstract] OR “Machine Predictive Model”[Title/Abstract] OR “Factor Risk”[Title/Abstract] OR “Risk Factor”[Title/Abstract] OR “Population at Risk”[Title/Abstract] OR “Populations at Risk”[Title/Abstract] OR “Clinical Decision Rule”[Title/Abstract] OR “Decision Rule Clinical”[Title/Abstract] OR “Decision Rules Clinical”[Title/Abstract] OR “Clinical Prediction Rule”[Title/Abstract] OR “Nomogram”[Title/Abstract] OR “Partin Tables”[Title/Abstract] OR “Partin Table”[Title/Abstract] OR “Table Partin”[Title/Abstract] OR “Risk prediction”[Title/Abstract] OR “Predict”[Title/Abstract] OR “prognostic model”[Title/Abstract] OR “Prediction model”[Title/Abstract] OR “Estimate”[Title/Abstract] OR “Area under the curve”[Title/Abstract] OR “Stratification” [Title/Abstract] OR “Discrimination” [Title/Abstract] OR “Discriminate” [Title/Abstract] OR “C-statistic” [Title/Abstract] OR “C statistic” [Title/Abstract] OR “AUC” [Title/Abstract] OR “Calibration” [Title/Abstract] OR “Indices” [Title/Abstract] OR “Algorithm” [Title/Abstract] OR “Multivariable”[Title/Abstract]
3. Outcome	AND	
	Subject Headings	“Compassion Fatigue”[MeSH Terms]
	Keywords	“Fatigue Compassion”[Title/Abstract] OR “Secondary Trauma”[Title/Abstract] OR “Secondary Traumas”[Title/Abstract] OR “Trauma Secondary”[Title/Abstract] OR “Traumas Secondary”[Title/Abstract] OR “Secondary Traumatization”[Title/Abstract] OR “Secondary Traumatizations”[Title/Abstract] OR “Traumatization Secondary”[Title/Abstract] OR “Traumatizations Secondary”[Title/Abstract] OR “Vicarious Trauma”[Title/Abstract] OR “Traumas Vicarious”[Title/Abstract] OR “Trauma Vicarious”[Title/Abstract] OR “Vicarious Traumas”[Title/Abstract] OR “Secondary Traumatic Stress”[Title/Abstract] OR “Stresses Secondary Traumatic”[Title/Abstract] OR “Stress Secondary Traumatic”[Title/Abstract] OR “Traumatic Stress Secondary”[Title/Abstract] OR “Vicarious Traumatization”[Title/Abstract] OR “Traumatization Vicarious”[Title/Abstract]

### Data collection and analysis

#### Data management.

All records retrieved from the databases will be imported into EndNote X9 for management. Duplicates will then be identified and removed using EndNote’s duplicate-detection function.

### Selection process

**Fig 1:**
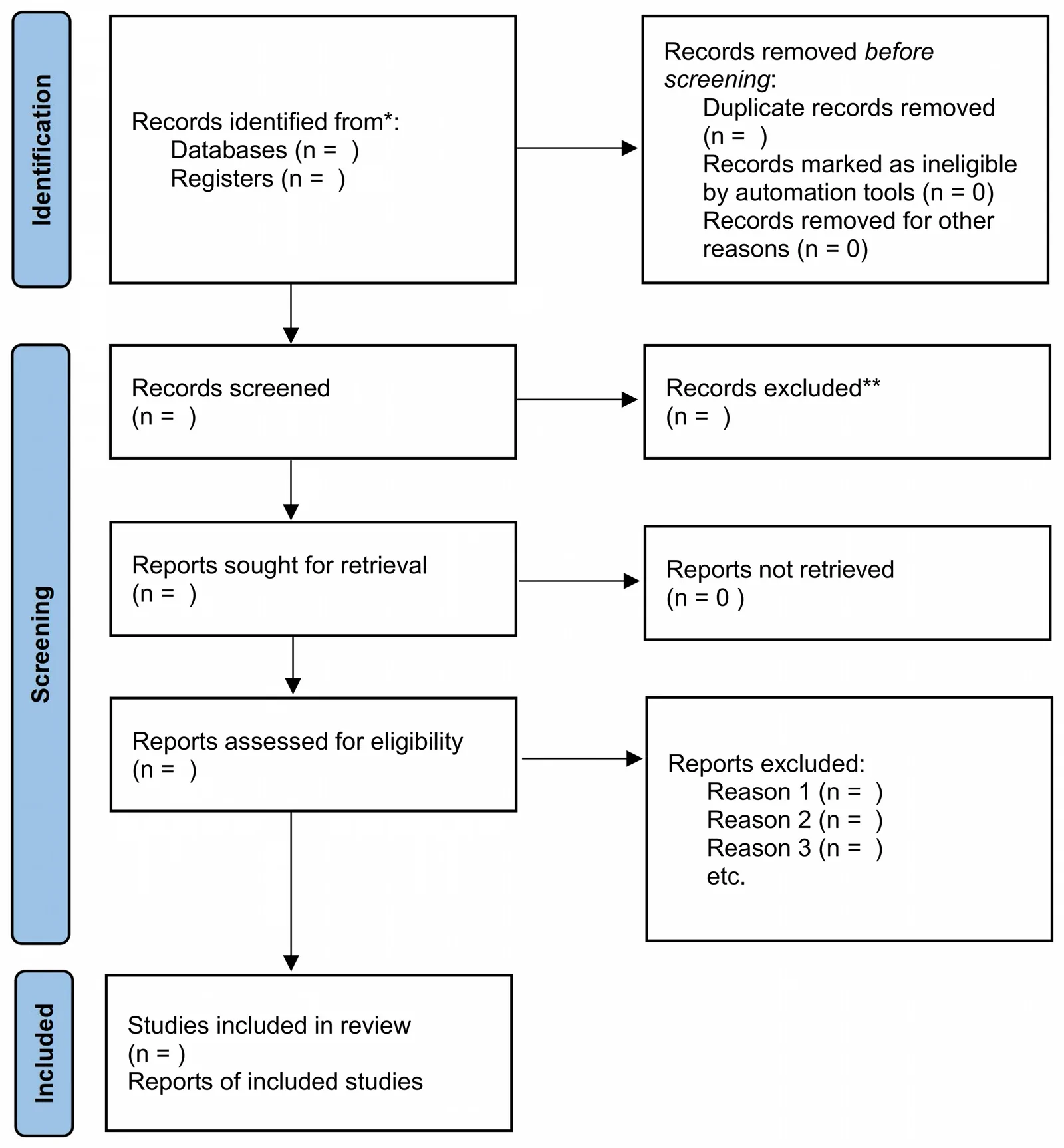
Flow chart of study selection.

We will use dual independent screening to ensure the accuracy and reliability of study selection. Two reviewers with nursing expertise will independently screen the titles and abstracts of all retrieved records against prespecified eligibility criteria. Records deemed potentially relevant or with uncertain eligibility will be carried forward to full-text assessment. The same two reviewers will then independently assess the full texts of the retained articles. Any disagreements will be resolved through discussion and, if necessary, adjudication by a third reviewer. The study selection process will be presented in a PRISMA flow diagram (Fig 1).

### Data extraction

Two researchers will independently extract data using a standardized data extraction form (e.g., in Microsoft Excel) and will then cross-check all extracted information to ensure accuracy. Any discrepancies or inconsistencies will be resolved by re-examining the source articles and reaching a consensus.

The data extraction table was developed with reference to the CHARMS checklist and comprises 11 fields, including study objective, data source, participants, predicted outcome(s), candidate predictors, and other relevant information [27]. Model performance measures will include calibration and discrimination, as well as Decision Curve Analysis (DCA). DCA facilitates identification of the optimal clinical decision strategy by visually comparing net benefit across a range of threshold probabilities [34,35].

Because compassion fatigue is assessed using standardized scales, we will extract both continuous compassion fatigue scores and the prevalence (i.e., the proportion of participants classified as having compassion fatigue) within each sample. We will also extract the criteria used to define compassion fatigue (e.g., for the Chinese version of the ProQOL, compassion fatigue will be described if any one of the three dimensions—compassion satisfaction (<37 points), occupational burnout (>27 points), or secondary traumatic stress/emergency response (>27 points)—meets the specified threshold).

### Critical appraisal

Two reviewers will independently assess the risk of bias and applicability of the included studies using the Prediction model Risk Of Bias Assessment Tool (PROBAST) [28]. Assessments will be performed in accordance with the PROBAST guidance, including evaluation of relevant signalling questions and assignment of domain-level judgments. Risk of bias will be assessed across four domains: participants, predictors, outcome, and analysis, whereas applicability will be assessed across three domains: participants, predictors, and outcome.

For each domain, risk of bias and applicability concerns will be rated as low, high, or unclear, as appropriate. An overall risk-of-bias judgment will be assigned to each prediction model in accordance with the PROBAST recommendations. If a study reports multiple prediction models, external validations, outcomes, time points, or analytical approaches, each eligible prediction model or validation analysis will be assessed separately. Decisions regarding which model, outcome, time point, or analysis to extract and appraise will be prespecified according to the eligibility criteria and data-extraction plan, rather than based on the reported results.

Disagreements between the two reviewers will be resolved through discussion and, when necessary, adjudication by a third reviewer. To minimize the risk of selective analytical decisions and potential bias, all appraisal rules and any deviations from the planned procedures will be documented. Assessment results will be summarized in tables that present domain-level and overall judgments on risk of bias and applicability.

### Qualitative data synthesis of prediction models

Extracted data will be presented in tabular format, summarizing study characteristics, participant demographics, and model details. Descriptive analyses will be performed on the prevalence of compassion fatigue (mean scores and prevalence rates), the frequency of reported predictors, and model performance metrics. Performance metrics will include discrimination (e.g., sensitivity, specificity, AUC, R^2^), calibration (calibration plots and Hosmer–Lemeshow test results), and clinical utility (decision curve analysis, DCA). Given the anticipated heterogeneity in model types (e.g., logistic regression for binary outcomes vs. linear regression for continuous scores) and study settings, a meta-analysis is unlikely to be feasible. To account for this, we will conduct a structured narrative synthesis. Studies will be grouped by key characteristics—including clinical setting, sample size, and validation status—to compare findings across these categories. Within each group, findings will be synthesized descriptively to explore patterns and potential sources of heterogeneity. Compassion fatigue, burnout, and STS will be considered the three outcomes of this study and reported separately, in line with the analytic approach described above.

### Reporting and presentation of findings

The systematic review will follow the PRISMA 2020 statement [29]. Upon completion, all extracted datasets, assessment tables, and supporting materials will be made publicly available as supplementary materials with the final publication.

## Discussion and conclusion

Although relevant reviews of compassion fatigue are available, they mainly focus on definitions, associated factors, and interventions among nurses [10,36,37]. Our preliminary review suggests that existing prediction models for compassion fatigue in nurses report inconsistent predictors and performance [23–25]. This systematic review will comprehensively summarize these published prediction models. By synthesizing model characteristics, candidate predictors, predictive performance, and related metrics, this review may assist nursing managers. It may support early identification of compassion fatigue and timely implementation of targeted interventions among nurses.

### Strengths and limitations

A key strength of this study is that it systematically reviews existing prediction models for compassion fatigue in nurses, describing their model characteristics, predictors, predictive performance, and other relevant metrics. This review may help nursing managers select appropriate prediction models and serve as a reference for future research on models for compassion fatigue. However, this study has limitations. Owing to differences in nurses’ baseline characteristics across studies and the inconsistent use of compassion fatigue measurement tools, substantial heterogeneity was present. Therefore, we did not undertake a meta-analysis; we conducted only a descriptive synthesis.

## Supporting information

S1 Appendix Preferred Reporting Items for Systematic Review and Meta -Analysis Protocols (PRISMA-P). (PDF)

S2 Appendix Critical Appraisal and Data Extraction for Systematic Reviews of Prediction Modeling Studies (CHARMS). (PDF)

S3 Appendix Prediction Model Risk of Bias Assessment Tool (PROBAST). (PDF)

S4 Appendix Preferred Reporting Items for Systematic Reviews and Meta-Analyses (PRISMA) 2020 statement. (PDF)

S5 Appendix Search strategy. (DOCX)
