# Supplementary material for: Prediction models for compassion fatigue in nurses: A protocol for systematic review and critical appraisal
Source: PLoS One. 2026 Sep 15;21(9):e0342524. doi: 10.1371/journal.pone.0342524 (PMC13577402; doi:10.1371/journal.pone.0342524)
Supplement: Supplementary file 2 [file pone.0342524.s002.pdf]

Item	Comments and examples
**1. Prognostic versus diagnostic prediction model**	Define whether the aim is to review models to predict:
	• Future events: prognostic prediction models
	• Current (disease) status: diagnostic prediction models
**2. Intended scope of the review**	Define intended scope of the review and intended purpose of the models reviewed in it. Examples:
	• Models to inform physicians' therapeutic decision making
	• Models to inform referral to or withholding from invasive diagnostic testing
**3. Type of prediction modelling studies (see also Box 1)**	Define the type of prediction modelling studies to include. Examples of study types (Box 1):
	• Prediction model development without external validation in independent data
	• Prediction model development with external validation in independent data
	• External model validation, possibly with model updating
**4. Target population to whom the prediction model applies**	Define the target population relevant to the review scope. Examples:
	• Women with diagnosed breast cancer
	• Healthy adult men in the general population
**5. Outcome to be predicted**	Define the outcome of interest to be predicted:
	• Specific future event, such as a fatal or non-fatal coronary heart disease
	• Specific diagnostic target disease, such as presence of lung embolism
**6. Time span of prediction**	Define over what specific time period the outcome is predicted (prognostic models only). Example:
	• Event within a specific time interval, such as event within 3 months, 1 year, or 10 years
**7. Intended moment of using the model**	The systematic review may focus on models to be used at a specific moment in time. Examples:
	• Models to be used at the moment of diagnosis of a particular disease
	• Models to be used preoperatively to predict the risk of postoperative complications
	• Models to be used in asymptomatic adults to detect undiagnosed type 2 diabetes mellitus

doi:10.1371/journal.pmed.1001744.t001

Domain	Key items	General	Applicability	Risk of bias
**Source of data**	• Source of data (e.g., cohort, case-control, randomised trial participants, or registry data)		X	X
**Participants**	• Participant eligibility and recruitment method (e.g., consecutive participants, location, number of centres, setting, inclusion and exclusion criteria)	X	X	
	• Participant description	X	X	
	• Details of treatments received, if relevant		X	X
	• Study dates	X	X	
**Outcome(s) to be predicted**	• Definition and method for measurement of outcome		X	X
	• Was the same outcome definition (and method for measurement) used in all patients?			X
	• Type of outcome (e.g., single or combined endpoints)	X	X	
	• Was the outcome assessed without knowledge of the candidate predictors (i.e., blinded)?			X
	• Were candidate predictors part of the outcome (e.g., in panel or consensus diagnosis)?			X
	• Time of outcome occurrence or summary of duration of follow-up		X	
**Candidate predictors (or index tests)**	• Number and type of predictors (e.g., demographics, patient history, physical examination, additional testing, disease characteristics)	X		
	• Definition and method for measurement of candidate predictors		X	X
	• Timing of predictor measurement (e.g., at patient presentation, at diagnosis, at treatment initiation)		X	
	• Were predictors assessed blinded for outcome, and for each other (if relevant)?			X
	• Handling of predictors in the modelling (e.g., continuous, linear, non-linear transformations or categorised)			X
**Sample size**	• Number of participants and number of outcomes/events	X		
	• Number of outcomes/events in relation to the number of candidate predictors (Events Per Variable)			X
**Missing data**	• Number of participants with any missing value (include predictors and outcomes)	X		X
	• Number of participants with missing data for each predictor			X
	• Handling of missing data (e.g., complete-case analysis, imputation, or other methods)			X
**Model development**	• Modelling method (e.g., logistic, survival, neural networks, or machine learning techniques)	X		
	• Modelling assumptions satisfied			X
	• Method for selection of predictors for inclusion in multivariable modelling (e.g., all candidate predictors, pre-selection based on unadjusted association with the outcome)			X
	• Method for selection of predictors during multivariable modelling (e.g., full model approach, backward or forward selection) and criteria used (e.g., p-value, Akaike Information Criterion)			X
	• Shrinkage of predictor weights or regression coefficients (e.g., no shrinkage, uniform shrinkage, penalized estimation)		X	X
**Model performance**	• Calibration (calibration plot, calibration slope, Hosmer-Lemeshow test) and Discrimination (C-statistic, D-statistic, log-rank) measures with confidence intervals		X	
	• Classification measures (e.g., sensitivity, specificity, predictive values, net reclassification improvement) and whether a priori cut points were used			X
**Model evaluation**	• Method used for testing model performance: development dataset only (random split of data, resampling methods, e.g., bootstrap or cross-validation, none) or separate external validation (e.g., temporal, geographical, different setting, different investigators)			X
	• In case of poor validation, whether model was adjusted or updated (e.g., intercept recalibrated, predictor effects adjusted, or new predictors added)		X	X
**Results**	• Final and other multivariable models (e.g., basic, extended, simplified) presented, including predictor weights or regression coefficients, intercept, baseline survival, model performance measures (with standard errors or confidence intervals)	X	X	
	• Any alternative presentation of the final prediction models, e.g., sum score, nomogram, score chart, predictions for specific risk subgroups with performance	X	X	
	• Comparison of the distribution of predictors (including missing data) for development and validation datasets			X
**Interpretation and Discussion**	• Interpretation of presented models (confirmatory, i.e., model useful for practice versus exploratory, i.e., more research needed)	X	X	
	• Comparison with other studies, discussion of generalizability, strengths and limitations	X	X	

doi:10.1371/journal.pmed.1001744.t002
