# Supplementary material for: Prediction models for compassion fatigue in nurses: A protocol for systematic review and critical appraisal
Source: PLoS One. 2026 Sep 15;21(9):e0342524. doi: 10.1371/journal.pone.0342524 (PMC13577402; doi:10.1371/journal.pone.0342524)
Supplement: Supplementary file 3 [file pone.0342524.s003.pdf]

Table 1. Four Steps in PROBAST

Step	Task	When to Complete
1	Specify your systematic review question(s)	Once per systematic review
2	Classify the type of prediction model evaluation	Once for each model of interest in each publication being assessed, for each relevant outcome
3	Assess risk of bias and applicability (per domain)	Once for each development and validation of each distinct prediction model in a publication
4	Overall judgment of risk of bias and applicability	Once for each development and validation of each distinct prediction model in a publication

PROBAST = Prediction model Risk Of Bias ASsessment Tool.

Table 2. PROBAST: Summary of Step 3—Assessment of Risk of Bias and Concerns Regarding Applicability*

1. Participants	2. Predictors	3. Outcome	4. Analysis
**Signaling questions**			
1.1. Were appropriate data sources used, e.g., cohort, RCT, or nested case–control study data?	2.1. Were predictors defined and assessed in a similar way for all participants?	3.1. Was the outcome determined appropriately?	4.1. Were there a reasonable number of participants with the outcome?
1.2. Were all inclusions and exclusions of participants appropriate?	2.2. Were predictor assessments made without knowledge of outcome data?	3.2. Was a prespecified or standard outcome definition used?	4.2. Were continuous and categorical predictors handled appropriately?
–	2.3. Are all predictors available at the time the model is intended to be used?	3.3. Were predictors excluded from the outcome definition?	4.3. Were all enrolled participants included in the analysis?
–	–	3.4. Was the outcome defined and determined in a similar way for all participants?	4.4. Were participants with missing data handled appropriately?
–	–	3.5. Was the outcome determined without knowledge of predictor information?	4.5. Was selection of predictors based on univariable analysis avoided?†
–	–	3.6. Was the time interval between predictor assessment and outcome determination appropriate?	4.6. Were complexities in the data (e.g., censoring, competing risks, sampling of control participants) accounted for appropriately?
–	–	–	4.7. Were relevant model performance measures evaluated appropriately?
–	–	–	4.8. Were model overfitting, underfitting, and optimism in model performance accounted for?†
–	–	–	4.9. Do predictors and their assigned weights in the final model correspond to the results from the reported multivariable analysis?†
**ROB**			
Selection of participants	Predictors or their assessment	Outcome or its determination	Analysis
**Applicability**			
Included participants or setting does not match the review question	Definition, assessment, or timing of predictors does not match the review question	Its definition, timing, or determination does not match the review question	–

RCT = randomized controlled trial; ROB = risk of bias.
* For further details, please see the explanation and elaboration document (27), available at Annals.org, and www.probast.org. Signaling questions are answered as yes, probably yes, probably no, no, or no information. ROB and concerns for applicability are rated as low, high, or unclear.
† Development studies only.

Table 3. Suggested Tabular Presentation for PROBAST Results*

Study	ROB				Applicability			Overall	
	Participants	Predictors	Outcome	Analysis	Participants	Predictors	Outcome	ROB	Applicability
1	+	–	?	+	+	+	+	–	+
2	+	+	+	+	+	+	+	+	+
3	+	+	+	?	–	+	+	?	–
4	–	?	?	–	+	+	–	–	–
5	+	+	+	+	+	?	+	+	?
6	+	+	+	+	?	+	?	+	?
7	?	?	+	?	+	+	+	?	+
8	+	+	+	+	+	+	+	+	+

PROBAST = Prediction model Risk Of Bias ASsessment Tool; ROB = risk of bias.
* + indicates low ROB/low concern regarding applicability; – indicates high ROB/high concern regarding applicability; and ? indicates unclear ROB/unclear concern regarding applicability.
