# Supplementary material for: Prediction models for compassion fatigue in nurses: A protocol for systematic review and critical appraisal
Source: PLoS One. 2026 Sep 15;21(9):e0342524. doi: 10.1371/journal.pone.0342524 (PMC13577402; doi:10.1371/journal.pone.0342524)
Supplement: Supplementary file 5 [file pone.0342524.s005.docx]

**1. PubMed**

**#1:** "nurses"[MeSH Terms] OR "nursing"[MeSH Terms]

**#2:** "nursing personnel"[Title/Abstract] OR "personnel nursing"[Title/Abstract] OR "Nurse"[Title/Abstract] OR "Nursings"[Title/Abstract]

**#3:**  #1 OR #2

**#4:** "predictive learning models"[MeSH Terms] OR "risk factors"[MeSH Terms] OR "clinical decision rules"[MeSH Terms] OR "nomograms"[MeSH Terms] OR "roc curve"[MeSH Terms] OR "predictive value of tests"[MeSH Terms] OR "observer variation"[MeSH Terms]

**#5:** "Learning Model Predictive"[Title/Abstract] OR"Model Predictive Learning"[Title/Abstract] OR"Predictive Learning Model"[Title/Abstract] OR"Predictive Models Machine"[Title/Abstract] OR"Machine Predictive Model"[Title/Abstract] OR"Factor Risk"[Title/Abstract] OR "Risk Factor"[Title/Abstract] OR "Population at Risk"[Title/Abstract] OR "Populations at Risk"[Title/Abstract] OR "Clinical Decision Rule"[Title/Abstract] OR "Decision Rule Clinical"[Title/Abstract] OR "Decision Rules Clinical"[Title/Abstract] OR "Clinical Prediction Rule"[Title/Abstract] OR "Nomogram"[Title/Abstract] OR "Partin Tables"[Title/Abstract] OR "Partin Table"[Title/Abstract] OR "Table Partin"[Title/Abstract] OR "Risk prediction"[Title/Abstract] OR "Predict"[Title/Abstract] OR"prognostic model"[Title/Abstract] OR"Prediction model"[Title/Abstract] OR "Estimate"[Title/Abstract] OR"Area under the curve"[Title/Abstract] OR"Stratification" [Title/Abstract] OR "Discrimination" [Title/Abstract] OR "Discriminate" [Title/Abstract] OR "C-statistic" [Title/Abstract] OR "C statistic" [Title/Abstract] OR "AUC" [Title/Abstract] OR "Calibration" [Title/Abstract] OR "Indices" [Title/Abstract] OR "Algorithm" [Title/Abstract] OR"Multivariable"[Title/Abstract]

**#6:**  #4 OR #5

**#7:** "compassion fatigue"[MeSH Terms]

**#8:** "Fatigue Compassion"[Title/Abstract] OR"Secondary Trauma"[Title/Abstract] OR"Secondary Traumas"[Title/Abstract] OR"Trauma Secondary"[Title/Abstract] OR"Traumas Secondary"[Title/Abstract] OR"Secondary Traumatization"[Title/Abstract] OR"Secondary Traumatizations"[Title/Abstract] OR"Traumatization Secondary"[Title/Abstract] OR"Traumatizations Secondary"[Title/Abstract] OR"Vicarious Trauma"[Title/Abstract] OR"Traumas Vicarious"[Title/Abstract] OR"Trauma Vicarious"[Title/Abstract] OR"Vicarious Traumas"[Title/Abstract] OR"Secondary Traumatic Stress"[Title/Abstract] OR"Stresses Secondary Traumatic"[Title/Abstract] OR"Stress Secondary Traumatic"[Title/Abstract] OR"Traumatic Stress Secondary"[Title/Abstract] OR"Vicarious Traumatization"[Title/Abstract] OR"Traumatization Vicarious"[Title/Abstract]

**#9:**  #7 OR #8

**#10:**  #3 AND #6 AND #9

**2.Web of Science**

**#1:** TS=("nurses" or "nursing")

**#2:** TS=("nursing personnel" OR "personnel nursing" OR "Nurse" OR "Nursings")

**#3:**  #1 OR #2

**#4:** TS=("predictive learning models" OR "risk factors" OR "clinical decision rules" OR "nomograms" OR "roc curve" OR "predictive value of tests" OR "observer variation")

**#5:** TS=("Learning Model Predictive" OR"Model Predictive Learning" OR"Predictive Learning Model" OR"Predictive Models Machine" OR"Machine Predictive Model" OR"Factor Risk" OR "Risk Factor" OR "Population at Risk" OR "Populations at Risk" OR "Clinical Decision Rule" OR "Decision Rule Clinical" OR "Decision Rules Clinical" OR "Clinical Prediction Rule" OR "Nomogram" OR "Partin Tables" OR "Partin Table" OR "Table Partin" OR "Risk prediction" OR "Predict" OR"prognostic model" OR"Prediction model" OR "Estimate" OR"Area under the curve" OR"Stratification" OR "Discrimination" OR "Discriminate" OR "C-statistic" OR "C statistic" OR "AUC" OR "Calibration" OR "Indices" OR "Algorithm" OR"Multivariable")

**#6:**  #4 OR #5

**#7:** TS=("compassion fatigue")

**#8:** TS=("Fatigue Compassion" OR"Secondary Trauma" OR"Secondary Traumas" OR"Trauma Secondary" OR"Traumas Secondary" OR"Secondary Traumatization" OR"Secondary Traumatizations" OR"Traumatization Secondary" OR"Traumatizations Secondary" OR"Vicarious Trauma" OR"Traumas Vicarious" OR"Trauma Vicarious" OR"Vicarious Traumas" OR"Secondary Traumatic Stress" OR"Stresses Secondary Traumatic" OR"Stress Secondary Traumatic" OR"Traumatic Stress Secondary" OR"Vicarious Traumatization" OR"Traumatization Vicarious")

**#9:**  #7 OR #8

**#10:**  #3 AND #6 AND #9

**3.The Cochrane Library**

**#1:** MeSH descriptor: [Nursing] explode all trees

**#2:** MeSH descriptor: [Nursing] explode all trees

**#3:** ("nursing personnel" OR "personnel nursing" OR "Nurse" OR "Nursings"):ti,ab,kw

**#4:** #1 OR #2 OR #3

**#5:** MeSH descriptor: [Predictive learning models] explode all trees

**#6:** MeSH descriptor: [Risk factors] explode all trees

**#7:** MeSH descriptor: [Clinical decision rules] explode all trees

**#8:** MeSH descriptor: [Nomograms] explode all trees

**#9:** MeSH descriptor: [Roc curve] explode all trees

**#10:** MeSH descriptor: [Predictive value of tests] explode all trees

**#11:** MeSH descriptor: [Observer variation] explode all trees

**#12:** ("Learning Model Predictive" OR"Model Predictive Learning" OR"Predictive Learning Model" OR"Predictive Models Machine" OR"Machine Predictive Model" OR"Factor Risk" OR "Risk Factor" OR "Population at Risk" OR "Populations at Risk" OR "Clinical Decision Rule" OR "Decision Rule Clinical" OR "Decision Rules Clinical" OR "Clinical Prediction Rule" OR "Nomogram" OR "Partin Tables" OR "Partin Table" OR "Table Partin" OR "Risk prediction" OR "Predict" OR"prognostic model" OR"Prediction model" OR "Estimate" OR"Area under the curve" OR"Stratification" OR "Discrimination" OR "Discriminate" OR "C-statistic" OR "C statistic" OR "AUC" OR "Calibration" OR "Indices" OR "Algorithm" OR"Multivariable"):ti,ab,kw

**#13:** #5 OR #6 OR #7 OR #8 OR #9 OR #10 OR #11 OR #12

**#14:** MeSH descriptor: [Compassion fatigue] explode all trees

**#15:** ("Fatigue Compassion" OR"Secondary Trauma" OR"Secondary Traumas" OR"Trauma Secondary" OR"Traumas Secondary" OR"Secondary Traumatization" OR"Secondary Traumatizations" OR"Traumatization Secondary" OR"Traumatizations Secondary" OR"Vicarious Trauma" OR"Traumas Vicarious" OR"Trauma Vicarious" OR"Vicarious Traumas" OR"Secondary Traumatic Stress" OR"Stresses Secondary Traumatic" OR"Stress Secondary Traumatic" OR"Traumatic Stress Secondary" OR"Vicarious Traumatization" OR"Traumatization Vicarious"):ti,ab,kw

**#16:** #14 OR #15

**#17:** #4 AND #13 AND #16

**4.Embase**

**#1: (**nurses OR nursing):ti,ab,kw

**#2:** (nursing personnel OR personnel nursing OR Nurse OR Nursings):ti,ab,kw

**#3:**  #1 OR #2

**#4:** (predictive learning models OR risk factors OR clinical decision rules OR nomograms OR roc curve OR predictive value of tests OR observer variation):ti,ab,kw

**#5:** (Learning Model Predictive ORModel Predictive Learning ORPredictive Learning Model ORPredictive Models Machine ORMachine Predictive Model ORFactor Risk OR Risk Factor OR Population at Risk OR Populations at Risk OR Clinical Decision Rule OR Decision Rule Clinical OR Decision Rules Clinical OR Clinical Prediction Rule OR Nomogram OR Partin Tables OR Partin Table OR Table Partin OR Risk prediction OR Predict ORprognostic model ORPrediction model OR Estimate ORArea under the curve ORStratification OR Discrimination OR Discriminate OR C-statistic OR C statistic OR AUC OR Calibration OR Indices OR Algorithm ORMultivariable):ti,ab,kw

**#6:**  #4 OR #5

**#7:** (compassion fatigue):ti,ab,kw

**#8:** (Fatigue Compassion OR Secondary Trauma OR Secondary Traumas OR Trauma Secondary OR Traumas Secondary OR Secondary Traumatization OR Secondary Traumatizations OR Traumatization Secondary OR Traumatizations Secondary OR Vicarious Trauma OR Traumas Vicarious OR Trauma Vicarious OR Vicarious Traumas OR Secondary Traumatic Stress OR Stresses Secondary Traumatic OR Stress Secondary Traumatic OR Traumatic Stress Secondary OR Vicarious Traumatization OR Traumatization Vicarious):ti,ab,kw

**#9:**  #7 OR #8

**#10:**  #3 AND #6 AND #9

**5.CINAHL**

**#1:** AB **(**nurses OR nursing)

**#2:** AB (nursing personnel OR personnel nursing OR Nurse OR Nursings)

**#3:**  #1 OR #2

**#4:** AB (predictive learning models OR risk factors OR clinical decision rules OR nomograms OR roc curve OR predictive value of tests OR observer variation)

**#5:** AB (Learning Model Predictive ORModel Predictive Learning ORPredictive Learning Model ORPredictive Models Machine ORMachine Predictive Model ORFactor Risk OR Risk Factor OR Population at Risk OR Populations at Risk OR Clinical Decision Rule OR Decision Rule Clinical OR Decision Rules Clinical OR Clinical Prediction Rule OR Nomogram OR Partin Tables OR Partin Table OR Table Partin OR Risk prediction OR Predict ORprognostic model ORPrediction model OR Estimate ORArea under the curve ORStratification OR Discrimination OR Discriminate OR C-statistic OR C statistic OR AUC OR Calibration OR Indices OR Algorithm ORMultivariable)

**#6:**  #4 OR #5

**#7:** AB (compassion fatigue)

**#8:** AB (Fatigue Compassion OR Secondary Trauma OR Secondary Traumas OR Trauma Secondary OR Traumas Secondary OR Secondary Traumatization OR Secondary Traumatizations OR Traumatization Secondary OR Traumatizations Secondary OR Vicarious Trauma OR Traumas Vicarious OR Trauma Vicarious OR Vicarious Traumas OR Secondary Traumatic Stress OR Stresses Secondary Traumatic OR Stress Secondary Traumatic OR Traumatic Stress Secondary OR Vicarious Traumatization OR Traumatization Vicarious)

**#9:**  #7 OR #8

**#10:**  #3 AND #6 AND #9

**6.PsycINFO**

**#1:** AB **(**nurses OR nursing)

**#2:** AB (nursing personnel OR personnel nursing OR Nurse OR Nursings)

**#3:**  #1 OR #2

**#4:** AB (predictive learning models OR risk factors OR clinical decision rules OR nomograms OR roc curve OR predictive value of tests OR observer variation)

**#5:** AB (Learning Model Predictive ORModel Predictive Learning ORPredictive Learning Model ORPredictive Models Machine ORMachine Predictive Model ORFactor Risk OR Risk Factor OR Population at Risk OR Populations at Risk OR Clinical Decision Rule OR Decision Rule Clinical OR Decision Rules Clinical OR Clinical Prediction Rule OR Nomogram OR Partin Tables OR Partin Table OR Table Partin OR Risk prediction OR Predict ORprognostic model ORPrediction model OR Estimate ORArea under the curve ORStratification OR Discrimination OR Discriminate OR C-statistic OR C statistic OR AUC OR Calibration OR Indices OR Algorithm ORMultivariable)

**#6:**  #4 OR #5

**#7:** AB (compassion fatigue)

**#8:** AB (Fatigue Compassion OR Secondary Trauma OR Secondary Traumas OR Trauma Secondary OR Traumas Secondary OR Secondary Traumatization OR Secondary Traumatizations OR Traumatization Secondary OR Traumatizations Secondary OR Vicarious Trauma OR Traumas Vicarious OR Trauma Vicarious OR Vicarious Traumas OR Secondary Traumatic Stress OR Stresses Secondary Traumatic OR Stress Secondary Traumatic OR Traumatic Stress Secondary OR Vicarious Traumatization OR Traumatization Vicarious)

**#9:**  #7 OR #8

**#10:**  #3 AND #6 AND #9

**7.CNKI**

**#1:** 篇关摘=护士OR护理人员OR护理工作者OR护理

**#2:** (篇关摘=预测模型OR风险预测OR模型OR预测OR列线图OR曲线下面积)

**#3:**  (篇关摘=同情疲劳OR共情疲劳OR同情疲乏OR同情心疲乏OR共情疲乏)

**#4:**  #1 AND #2 AND #3
